# Ten Years of 2D Longitudinal Strain for Early Myocardial Dysfunction Detection: A Clinical Overview

**DOI:** 10.1155/2018/8979407

**Published:** 2018-12-05

**Authors:** Concetta Zito, Luca Longobardo, Rodolfo Citro, Maurizio Galderisi, Lilia Oreto, Maria Ludovica Carerj, Roberta Manganaro, Maurizio Cusmà-Piccione, Maria Chiara Todaro, Gianluca Di Bella, Egidio Imbalzano, Bijoy K. Khandheria, Scipione Carerj

**Affiliations:** ^1^Department of Clinical and Experimental Medicine, Section of Cardiology, University of Messina, Messina, Italy; ^2^Department of Cardiology and Cardiac Surgery, University Hospital, San Giovanni di Dio, Salerno, Italy; ^3^Department of Advanced Biomedical Sciences, Federico II University Hospital, Naples, Italy; ^4^Department of Clinical and Experimental Medicine, Section of Internal Medicine, University of Messina, Messina, Italy; ^5^Aurora Cardiovascular Services, Aurora Sinai/Aurora St. Luke's Medical Centers, University of Wisconsin School of Medicine and Public Health, Marcus Family Fund for Echocardiography (ECHO) Research and Education, Milwaukee, WI, USA

## Abstract

In recent years, the role of left ventricular ejection fraction (EF) as the gold standard parameter for the evaluation of systolic function has been questioned, and many efforts have been concentrated in the clinical validation of new noninvasive tools for the study of myocardial contractility. Improvement in the accuracy of speckle-tracking echocardiography has resulted in a large amount of research showing the ability of two-dimensional strain to overcome EF limitations in the majority of primary and secondary heart diseases. Currently, global longitudinal strain (GLS) is considered the most accurate and sensitive parameter for the assessment of early left ventricular dysfunction. This review summarizes the advantages that this measurement can provide in several clinical settings. Moreover, the important cautions that should be considered in making the choice to use GLS also are addressed. Finally, a special focus on bull's-eye polar maps for the assessment of regional changes of longitudinal function and the usefulness of these maps in the differential diagnosis of several diseases is provided.

## 1. Two-Dimensional Longitudinal Strain: Introducing Concepts

The value of ejection fraction (EF) for the assessment of left ventricular (LV) function has been widely questioned during the past 10 to 15 years [1] because of intrinsic limitations, including late reduction only in an advanced stage of cardiovascular disease, poor reliability in patients with LV hypertrophy (LVH) and volume reduction, interobserver and intraobserver variability due to apical foreshortening, difficult endocardial border detection, etc.

With the potential to overcome these concerns, two-dimensional (2D) speckle-tracking echocardiography (STE) longitudinal strain was introduced. Global longitudinal strain (GLS) has been shown to be more reproducible and more useful clinically than circumferential and radial strains [2, 3]. It has been demonstrated to be as accurate as sonomicrometry and magnetic resonance imaging (MRI) in several conditions [4] and, ultimately, for predicting mortality in different clinical settings [5, 6]. Indeed, a recent meta-analysis that compared LVEF and GLS in predicting major adverse cardiac events in patients with different cardiovascular diseases reported that GLS had superior prognostic value to EF for predicting all-cause mortality, cardiac death, malignant arrhythmia, hospitalization due to heart failure, urgent valve surgery or heart transplantation, and acute coronary ischemic event [7].

**Figure 1:**
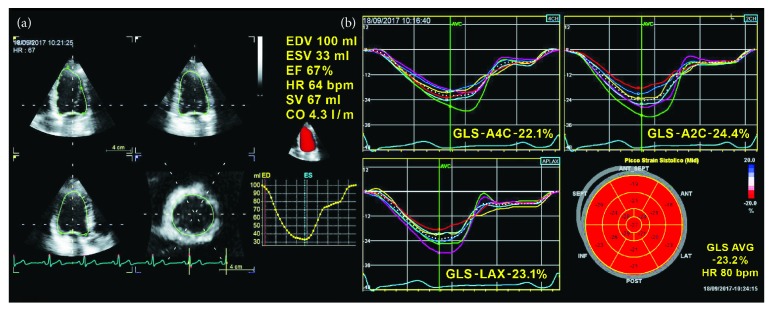
Echocardiographic assessment of left ventricular systolic function in a healthy subject through three-dimensional ejection fraction (a) and two-dimensional speckle-tracking echocardiography global longitudinal strain curves and bull's-eye map (b).

Moreover, the possibility of quantifying regional alterations of longitudinal strain (LS) through its polar projection, the so-called bull's-eye map, allows a further evaluation of both the site and extent of myocardial damage. This last analysis is particularly useful in visualizing regional inhomogeneities in function, in some cases providing the identification of typical pathological patterns with an incremental value for differential diagnosis (Figure 1).

General concepts about strain and STE and technical details about the practical performance of STE have been satisfactorily discussed elsewhere [8]; thus they will not be further analyzed in this review. Likewise, we will neither discuss the usefulness of LS in overt myocardial dysfunction nor the potential of 3D strain to overcome 2D GLS limitations. Although strain mechanics in other chambers (i.e., right ventricle, left atrium) provide important information, it is a broad topic that cannot be properly addressed in this context and has already been conveniently discussed [9, 10].

Therefore, the aim of this review is to offer a critical overview of 2D global and regional LS, underscoring the advantages and disadvantages of each for the early detection of myocardial dysfunction and whether strain should or should not be indicated in the clinical evaluation of the function (Supplemental Table 1). By reviewing studies available in the literature, we found that LS analysis seems to provide a relevant incremental value, and it should be applied especially in the following clinical conditions: (a) LVH and heart failure (HF) with preserved EF (HFpEF), (b) heart valve disease (HVD), (c) acute coronary syndrome and chronic ischemic cardiomyopathy, (d) acute myocarditis, (e) systemic and neuromuscular disorders, and (f) cardiotoxicity in oncologic patients.

### 1.1. Special Warnings and Precautions for Use

Before discussing the role of LS in several clinical settings, it is important to be aware of certain weaknesses that could affect the analysis (Table 1).

Reproducibility is the most important key point, and it could be affected by both clinical and technical issues.

Among the clinical issues, age and gender differences, hemodynamic factors, and volume status play the greatest roles. It recently was demonstrated that LS decreases with age and that men have lower LS values [3]. Moreover, in a porcine model it was shown that LS was significantly correlated with preload and afterload changes, limiting the ability to predict true inotropic function of the LV [11]. This could be particularly true in clinical conditions characterized by significant changes of load, such as aortic stenosis (AS) and patients treated by chemotherapy (who often are affected by vomiting and diarrhea), etc. Therefore, load dependency should be carefully considered, especially when serial evaluations of LS are performed. Stroke volume (SV) and heart rate also are variables that should be considered. The reduction of SV causes a reduction of strain values, and strain could appear low in pathological conditions with low SV, such as severe AS, even if myocardial contractility is preserved. Similarly, GLS increases in response to early physiological heart rate increase in the setting of exercise in normal patients but decreases in the setting of pathological heart rate increase, such as sepsis [12].

Technical issues affect LS even more frequently than clinical ones. Poor image quality reduces endocardial border and speckle detection, affecting LS reliability. Moreover, the skill level of the echocardiographer plays a pivotal role because a frame rate between 40 and 80 Hz, a careful placement of fiducial landmarks, an optimal apex visualization (avoiding foreshortened images), a correct setting of spatial and temporal smoothing, and a proper size of the region of interest significantly reduce strain variability. Thus, it is reasonable that strain analysis be performed by expert echocardiographers with specific training. In addition, variability increases in the regional distribution of LS, whereas the global estimation is less affected; thus, bull's-eye maps should be considered a valid tool for differential diagnosis only when image quality is optimal. Currently, it could be reasonable and more useful to consider regional longitudinal strain distribution to be a semiquantitative tool, focusing attention on the assessment of differences between segments in bull's-eye maps to find typical patterns that can help obtain the correct diagnosis, as opposed to evaluating the numerical segment-specific strain values, and comparing them in the follow-up. Finally, intervendor variability had been considered one of the most important technical limitations of strain analysis. However, important efforts have been made to reduce differences among vendors [13], and no intervendor differences for GLS were reported in a recent multicenter study [3].

These good results and the increased availability of strain technique determined an improved reproducibility of GLS, now reported to be analogous [14] or even better [2, 15] to that of LVEF. However, these issues affect regional strain more than global measurement, which should be kept in mind when this evaluation is performed.

## 2. LVH and HFpEF

**Figure 2:**
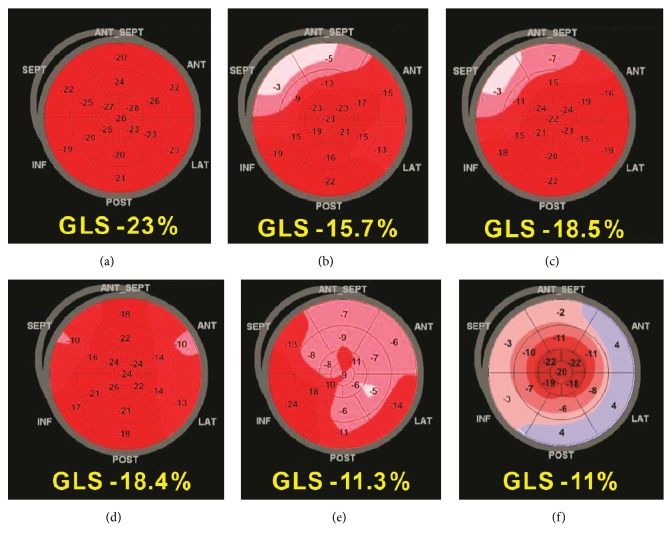
Bull's-eye maps of global longitudinal strain depicting different patterns of left ventricular hypertrophy: (a) athlete, (b) hypertension, (c) aortic stenosis with hypertrophy of basal segments, (d) heart failure with preserved ejection fraction and diffuse left ventricle hypertrophy, (e) hypertrophic cardiomyopathy, and (f) amyloidosis with classical “apical sparing” pattern.

LVH is perhaps the setting in which EF quantification fails most clearly in the detection of LV systolic function because LVH and volume changes allow depressed systolic function to go unnoticed when assessed by EF [16]. On the contrary, GLS provides additional information in the evaluation of LVH, and regional changes in LS seem to identify specific myocardial deformation patterns for some forms of myocardial hypertrophy (Figure 2 and Tables 2 and 3). Therefore, according to the evidence from previous studies and in our opinion, LS analysis is strongly indicated in this clinical scenario (Supplemental Table 1).

### 2.1. Athlete's Heart

Athlete's heart is a benign increase in cardiac mass that represents a physiological adaptation to systematic training. The most challenging clinical dilemma is the differentiation between this physiological LVH and hypertrophic cardiomyopathy (HCM), one of the common causes of sudden cardiac death in young athletes. EF is commonly normal in athletes [17]; however, it sometimes can be reduced at rest. Indeed, athletes generally have greater LV end-diastolic volume (LVEDV) because this is an adaptation to larger reserve and SV depends on heart rate. With a reduced heart rate, which is often seen in athletes at rest, the SV will be normal but EF may be reduced owing to a high LVEDV, despite normal LV contractility.

EF is often normal or supernormal in HCM patients as well, especially in the first stages of the disease [18]. On the other hand, GLS has been found to be normal or slightly reduced in athletes but significantly reduced in HCM patients [19, 20]. GLS reduction in athletes should not be considered the result of an impairment of myocardial contractility, but it could be a marker of a specific myocardial adaptation to the exercise-induced increase in volume overload, according to Laplace's law [20].

Moreover, analysis of the regional changes in LS might be suggested for differential diagnosis, given that LS is normal (with lower basal strain and more pronounced physiological base-to-apex gradient) in athletes (Figure 2(a)) but is often reduced in the segments more affected by pathological hypertrophy in HCM patients (Figure 2(e)). There is not a broad consensus about a well-defined cut-off value of regional LS and GLS to distinguish pathological and physiological hypertrophy in the literature. Caselli* et al*. [20] reported an average GLS value of -22% for athletes, but they did not compare them with subjects with pathological LVH. However, the evidence of extremely low segmental values (i.e., <-11%) definitively indicates HCM rather than physiological LVH.

### 2.2. Arterial Hypertension and Metabolic Disorders

In recent years, there has been much discussion about the role of GLS in the assessment of LV systolic function in hypertensive patients. Hypertension causes an increase in LV afterload. Because it is well known that LS is affected by LV overload, both preload and afterload, it is not completely clear if the reduction in GLS is associated with increased afterload or with the subendocardial ischemia and increased myocardial fibrosis that derive from it. Several studies have shown a significant reduction of GLS in hypertensive patients, both when they showed concentric LV remodeling [21] and when they did not [22] (Figure 2(b)). Interestingly, in the very first stages of hypertension, it is possible for a patient to show regional alterations while GLS remains in the normal values [23]. This would be a suggestive clue of the importance of the bull's-eye map in detecting segmental alterations of myocardial function despite normal global function.

Some recent studies have reported a reduction of GLS in patients with diabetes mellitus [24, 25], insulin resistance [26], or obesity [27], even though they had a normal EF. Mechanisms of cardiac involvement in these pathological conditions have not been completely clarified yet, but currently the development of LV remodeling and LVH associated with chamber stiffness are considered the main determinants of LV longitudinal performance impairment. However, again, the very high sensitivity of LS analysis is the counterpart to a low specificity, so it is frequently difficult to distinguish the real weight of the single pathological condition in patients who often have multiple conditions simultaneously (e.g., hypertension and diabetes mellitus).

### 2.3. HFpEF

Diabetes and hypertension, which are often associated with the impairment of longitudinal function, are also common findings in patients with HFpEF [21, 28, 29], and it is well known that LVH is the key structural change of this disease. Probably, repetitive ischemic insults due to macrovascular and microvascular abnormalities and interstitial fibrosis cause an early intrinsic depression of subendocardial longitudinal fiber contractility in these patients, especially in more hypertrophic hearts. For this reason, longitudinal myocardial performance is early impaired, whereas in the first stage of the disease the sparing of circumferential fibers results in EF remaining within the normal range [30] (Supplemental Figure 1). In addition, GLS has been shown to be a strong predictor of event-free survival [31], and it can be used to follow the progression of the disease because its decrease is strongly correlated with the increase of N-terminal probrain natriuretic peptide (NT-proBNP) and the onset of symptoms [31, 32]. Accordingly, the most recent European Society of Cardiology (ESC) guidelines on HF state that strain imaging should be considered in subjects at risk of developing overt HF in order to identify myocardial dysfunction at the preclinical stage (Class IIa, level of evidence C) [33].

### 2.4. Hypertrophic Cardiomyopathy

Sarcomeric and nonsarcomeric HCMs typically are characterized by heterogeneous echocardiographic patterns of LVH, often with a normal or supernormal EF. On the contrary, GLS provides an accurate estimation of LV contractility, and its reduction is associated with poor prognosis and increased risk of ventricular arrhythmias [34]. The role of regional distribution of LS impairment is fundamental in this setting. By looking at bull's-eye maps, it is possible to recognize extensive areas of severely reduced deformation with excellent discriminatory power for distinguishing sarcomeric HCM from hypertensive heart disease and other forms of LVH (the so-called phenocopies) (Figure 2(e)). Moreover, regional LS findings match the results of cardiac MRI, the gold standard for the assessment of wall thickness and regional fibrosis. Indeed, comparing late gadolinium enhancement areas of myocardial fibrosis by MRI to bull's-eye maps, LS was found to be significantly lower in segments with late gadolinium enhancement, suggesting that it can accurately identify myocardial fibrosis [35]. In addition, in hypertrophy-free HCM mutation carriers (Phe-/Gen+), it has been more recently demonstrated that regional LS is significantly impaired in the basal segments of the septum and that GLS is decreased compared with healthy controls [36].

### 2.5. HCM Phenocopies: Fabry Disease and Cardiac Amyloidosis

**Figure 3:**
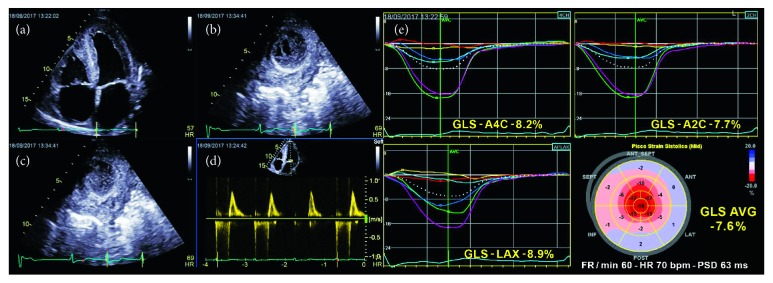
Clinical case of a 47-year-old man with amyloidosis. Please note left ventricular hypertrophy with paracardiac pathological tissue (Panels (a)-(c)) and diastolic dysfunction as assessed by mitral peak early (e) and late (a) velocities restrictive pattern (E/A > 2) with lowest, almost zero, A wave amplitude suggestive of atrial impairment (Panel (d)). Panel (e) shows left ventricular two-dimensional speckle-tracking echocardiography longitudinal strain curves and bull's-eye map with typical “apical sparing” pattern (Panel (e)).

The assessment of regional LS seems to be useful for the detection of some HCM phenocopies, such as Fabry disease and cardiac amyloidosis. In Fabry disease, regional systolic LS is typically decreased at the basal posterolateral wall (where replacement fibrosis usually is located), and a reduced LS in these segments indicates fibrosis, as confirmed by late gadolinium enhancement at MRI [37]. Also, the diagnosis of cardiac amyloidosis often is difficult because of the evidence of symmetric or asymmetric LVH with a normal EF, at least in the first stage of the disease. GLS usually is reduced in patients with cardiac amyloidosis, but it is in this setting that bull's-eye polar maps of LS show unmistakably their potential role in the differential diagnosis of HCM and other forms of LVH. Indeed, cardiac amyloidosis commonly is characterized by regional and progressive variations in LS from base to apex with a relative LV “apical sparing” pattern (Figures 2(f) and 3). Evidence of this typical pattern in the study of regional distribution of LS changes is easily recognizable and an accurate “red flag” for suspecting cardiac amyloidosis [24, 38, 39].

The equation

(1) relative  apical  LS=average  apical  LSaverage  basal  LS+average  mid  LS=1

obtained 93% sensitivity and 82% specificity in differentiating cardiac amyloidosis from other causes of LVH [40].

Of course, apical sparing pattern is not exclusive to cardiac amyloidosis and can be found in some other diseases, including LV noncompaction and AS. However, a careful standard echocardiographic examination would allow the physician to make a differential diagnosis with the help of other specific markers such as a typical appearance of the endocardium in LV noncompaction or valve calcifications in AS.

## 3. Heart Valve Disease

LVEF is a key parameter for establishing when an asymptomatic patient with severe heart valve disease (HVD) should be referred to surgery [41, 42]; however, EF sensitivity for the detection of myocardial dysfunction is lower than previously stated and EF changes occur late, when cardiac damage often is not reversible. GLS was tested for the assessment of all HVD. A reduced GLS is associated with aortic regurgitation (AR) and mitral regurgitation (MR) progression [43–45], and low values predict HF occurrence [46, 47] and impaired outcomes after surgery [43, 47]. Accordingly, the use of GLS for a more accurate evaluation of LV function in asymptomatic patients, particularly those with MR, has been suggested [48], but so far LS analysis for risk stratification of these patients has not been included in the guidelines for the management of HVD [49]. In our opinion, LS analysis remains a very useful option in these cases (Supplemental Table 1).

On the contrary, the most robust findings with regard to the usefulness of GLS have been obtained in patients with AS. GLS gradually decreases while AS severity increases without any simultaneous change in LVEF [50]. This finding could possibly be explained by load dependency of strain measurements; indeed, an increase in LV volumes determines a decrease in GLS, and that means that GLS decreases as AS gradient increases. However, the prognostic value of GLS is not reduced, as demonstrated by the evidence that, in asymptomatic patients, impaired GLS was associated with an increased risk of cardiac events over traditional risk markers—including EF and AS gradient [51–53]. Similar results have been obtained in asymptomatic patients with low-flow, low-gradient AS [54], in whom a reduced GLS was independently associated with mortality.

Moreover, GLS measured during dobutamine stress echocardiography may provide incremental prognostic value beyond GLS measured at rest and be helpful in enhancing risk stratification in low-flow, low-gradient AS [54].

Accordingly, the most recent multimodality imaging guidelines suggest GLS as a parameter for the assessment of risk in patients with asymptomatic severe AS, stating that surgery may be considered in patients with high risk (Class IIb) [55]. In addition, the prognostic role of GLS seems to be confirmed after transcatheter aortic valve replacement in high-risk patients [56].

Therefore, in our opinion, GLS is indicated for an accurate management of patients with severe AS (Supplemental Table 1). However, as previously discussed, LS is significantly affected by SV changes, being lower when SV is reduced; thus, the reduction of GLS in patients with severe AS could be partially influenced by the reduced SV and not a mere expression of LV systolic dysfunction. Thus, these concerns should be considered with extreme care when we include GLS in clinical decision-making regarding AS patients.

Regarding the role of regional distribution of LS, this shows a more significant worsening of longitudinal function at the basal segments of the anterior wall and septum (Figure 2(c) and Supplemental Figure 2) where a greater amount of fibrosis and hypertrophy is confirmed by MRI [57]. This pattern could help to correctly classify the LVH in those patients with AS and other comorbidities (e.g., hypertension, etc.), thereby improving their clinical management.

## 4. Acute Coronary Syndrome and Chronic Ischemic Cardiomyopathy

**Figure 4:**
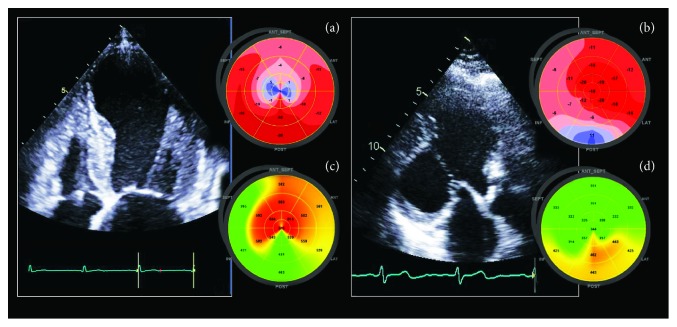
Echocardiographic assessment of postinfarction left ventricular remodeling through two-dimensional speckle-tracking echocardiography longitudinal strain bull's-eye maps in one patient with anterior myocardial infarction (a) and in another with inferior myocardial infarction (b). Red/blue bull's-eye maps (a, b) clearly identify regional alterations of longitudinal strain in the segments more affected by myocardial infarction (thin and dilated), whereas green/yellow bull's-eye maps (c, d) describe delayed activation (yellow and red areas) in the same segments, which can determine mechanical dispersion and increased risk of arrhythmias.

The assessment of LS is very useful (Supplemental Table 1) in the management of ischemic cardiomyopathy both as a diagnostic tool of acute coronary syndrome and in the postacute setting, where it provides important prognostic clues for predicting HF in patients with postinfarction LV remodeling (Figure 4 and Tables 2 and 3).

Regional LS seems to be more useful than GLS in this context because myocardial infarction usually affects circumscribed portions of myocardium. LS was shown to be more accurate than wall motion score index in identifying non-ST-elevation MI patients with acute coronary occlusion who may benefit from urgent reperfusion therapy [58]. Indeed, LS is the most sensitive tool for the detection of subendocardial fiber alterations, and because the subendocardium is the first myocardial layer that suffers from ischemia, regional LS analysis could be especially useful in those patients in whom epicardial fibers are preserved and mild wall motion changes could delay prompt revascularization [59]. Moreover, regional LS can improve the differential diagnosis between anterior MI and takotsubo syndrome, in which the circumferential pattern of the polar map is absolutely typical and quite different from that of anterior MI [60] or myocarditis (Supplemental Figure 3). However, it was reported recently that the accuracy of regional LS in identifying regional abnormality differed significantly among vendors in patients who had experienced a previous MI and that regional strain is more affected by a low reproducibility than GLS [2]. Thus, it would be reasonable to consider regional strain distribution as a semiquantitative tool with typical patterns that can help obtain the correct diagnosis, rather than evaluating the numerical segment-specific strain values and considering it to be the decisive tool for the diagnosis of infarction.

On the other hand, the prognostic importance of global longitudinal function assessment is well recognized. Indeed, a reduction in GLS seems to suggest an increased risk of death, reinfarction, congestive HF, or stroke [6] even more reliably than EF and wall motion score index changes [61].

**Figure 5:**
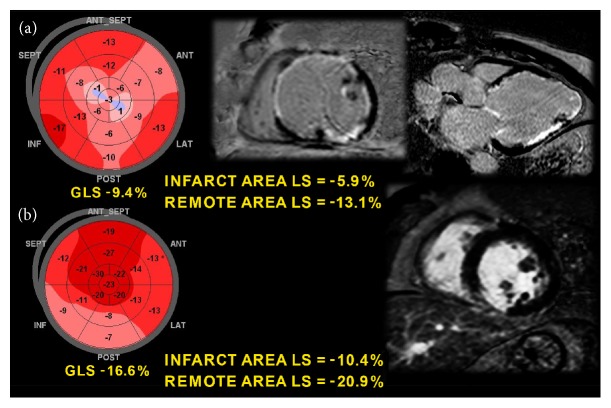
Comparison between global longitudinal strain and cardiac magnetic resonance imaging in two patients with (a) anterior myocardial infarction and (b) inferior myocardial infarction. The infarct areas of the bull's-eye map correspond closely with late gadolinium enhancement (scar) areas on magnetic resonance imaging.

A similar accuracy of MRI and regional LS has been reported in the assessment of both MI extension and transmurality in patients with STEMI [62] (Figure 5), in the detection of scar regions in candidates for cardiac resynchronization therapy [63], and in the identification of mechanical dispersion in post-MI patients with recurrent arrhythmias [64] (Figure 4). If these data are confirmed by further studies, regional and global LS assessment could be a potential alternative to MRI in these contexts, given that MRI is not always available, is more expensive, and is sometimes limited by the patient's clinical conditions.

Finally, with regard to stress echocardiography, the clinical use of GLS and regional LS is still limited; however, it has been demonstrated that GLS analysis provides an incremental value to wall motion analysis in the detection of significant coronary artery disease during dipyridamole and dobutamine stress echocardiography [65, 66].

## 5. Acute Myocarditis

**Figure 6:**
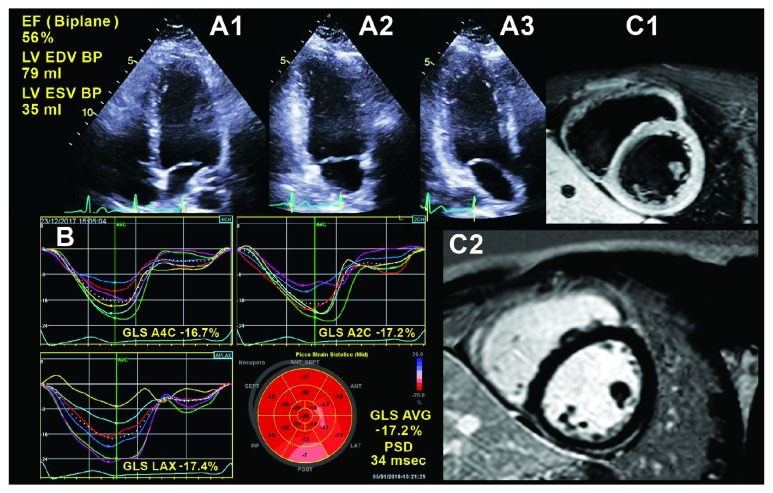
A 27-year-old male patient with myocarditis. Panel A1-3 shows normal left ventricular systolic function assessed by Simpson's method. Panel B shows left ventricular two-dimensional speckle-tracking echocardiography longitudinal strain curves and bull's-eye map: please note that the bull's-eye plot underlines regional alterations of longitudinal function in the inferolateral wall. In Panel C1-2, cardiac magnetic resonance shows a hyperenhancement area located in the epicardial layer of the inferior and inferolateral left ventricular walls, suggesting edema and fibrosis, respectively, on both T2 weighted and late gadolinium enhancement images; please note that bull's-eye data from two-dimensional speckle-tracking echocardiography are confirmed by the assessment of fibrosis in the same segments by cardiac magnetic resonance.

Acute myocarditis is characterized by inflammation of the myocardium as a result of exogenous or endogenous causes. The clinical diagnosis of acute myocarditis is based on symptoms, electrocardiography, elevated myocardial necrosis biomarkers, and echocardiography. Often, conventional echocardiography reveals no obvious changes in global cardiac function, and therefore, it has limited diagnostic value. Longitudinal systolic function commonly is affected in acute myocarditis (Supplemental Figure 3), so regional and global LS analysis provides additional fundamental information for the diagnosis [67]. In addition, GLS seems to be significantly correlated with the amount of myocardial edema, as recently confirmed in a series of patients with a cardiac MRI-verified diagnosis of acute myocarditis [68], providing an important support to clinical and conventional echocardiographic evaluation, especially in patients with preserved LVEF (Figure 6). Owing to these advantages, we believe that longitudinal strain analysis should be applied in all patients with suspicious or certain acute myocarditis.

## 6. Systemic Diseases and Neuromuscular Disorders

### 6.1. Systemic Diseases

In the definition of “systemic diseases,” we usually refer to a wide group of pathological conditions, sometimes autoimmune, characterized by the systemic involvement of the organism with various symptoms and often a progressive worsening of clinical status. A subtle myocardial dysfunction often occurs at both early and advanced stages of these diseases. Systemic sclerosis is one of the most common autoimmune systemic diseases that determine multiple cardiac abnormalities, including myocardial fibrosis and ischemia, which can lead to ventricular arrhythmias and both systolic and diastolic dysfunction. Cardiac fibrosis in these patients is related to recurrent vasospasm, poor vasodilator reserve, focal ischemia, and inflammation. However, early myocardial involvement is rarely highlighted by a reduced EF that becomes evident only in advanced stages [69]. Conversely, GLS, able to early detect myocardial dysfunction, is feasible and sensitive enough for the assessment of LV systolic dysfunction in these patients [69–71]. Moreover, GLS has been shown to be able to predict a reduced cardiopulmonary exercise capacity and the onset of ventricular arrhythmias [72], increasing its prognostic relevance. Similar results have been obtained in the evaluation of LV function by using GLS in patients with *β*-thalassemia major [73] and rheumatoid arthritis [74].

However, it is difficult in many cases to affirm whether a reduction in GLS is caused by the systemic disorder itself or is a consequence of other factors (e.g., medical therapy [azathioprine, methotrexate, cyclosporine A, etc.], as in systemic sclerosis patients) or associated comorbidities (hypertension and diabetes, mostly). So far, typical patterns of regional distribution of LS alterations have not been reported. Thus, further studies need to be performed to evaluate the effective role of GLS analysis in the follow-up of systemic disorders. Nowadays, this tool can be considered very useful but not indispensable for patient management (Supplemental Table 1).

### 6.2. Neuromuscular Disorders

Cardiac complications are the leading cause of death in patients with Duchenne muscular dystrophy (DMD). Echocardiography is the current standard for monitoring LV systolic function in these patients, but it might not detect early systolic dysfunction. Significant decreases in longitudinal peak systolic strain/strain rate and early diastolic myocardial velocities previously were found in the LV inferolateral and anterolateral walls in patients with DMD [75] (Supplemental Figure 4). More recently, it was shown that GLS is lower in DMD patients than controls [76], and bull's-eye maps clearly show the particular involvement of the inferolateral wall in these patients (Table 3; Supplemental Figure 4).

Interesting data have been reported about the role of 2D STE GLS in the assessment of subtle LV systolic dysfunction in patients with Friedreich's ataxia myocardial involvement, too. Friedreich's ataxia patients' hearts are characterized by iron deposits, diffuse fibrosis, focal necrosis, and LV hypertrophy, but often they show a normal LVEF and mass in the first stages of the disease [77–79]. However, it has been found that GLS was significantly reduced in patients compared with controls. Moreover, in patients treated with idebenone, GLS improvement preceded the reduction of hypertrophy and improvement of LVEF [80].

However, the clinical significance of the early detection of cardiac dysfunction in these diseases warrants further studies.

## 7. Cardiotoxicity from Cancer Therapy

The early recognition of myocardial damage in patients who undergo chemotherapy is crucial for their clinical management. Indeed, chemotherapy discontinuation is based on the assessment of myocardial dysfunction, thus far quantified by EF. The most recent position papers [81, 82] state that the diagnosis of cancer therapeutics-related cardiac dysfunction can be established if there is a decrease in LVEF of more than 10%, to a value less than 53%. However, an elegant study by Thavendiranathan* et al*. [83] showed that the interoperator variability of EF was about 10% in the assessment of LV systolic function in patients treated by chemotherapy, underlining the weakness of this measurement in this context. On the other hand, several studies confirmed the value of deformation imaging for early detection of LV dysfunction secondary to cancer therapy [84–86] (Table 2) and a systematic review reported the sensitivity of LS in the detection of subclinical LV dysfunction in patients treated with anthracyclines alone or in association with other therapies, either during treatment or late after completion of the therapy [87]. Moreover, it was shown that GLS reduction in patients treated with anthracycline or doxorubicin anticipates changes in LVEF [13], providing fundamental information for an early risk stratification of these subjects. On this basis, the same guidelines [81, 86] state that a relative percentage reduction in GLS of >15% from baseline should be considered abnormal and a marker of early LV subclinical dysfunction in patients treated by chemotherapy. However, at this time, a reduction in GLS is not indicated as a key parameter for therapy discontinuation because of the lack of randomized trials demonstrating that the GLS-oriented strategy can be superior to an EF-oriented strategy. Further, GLS accuracy is affected in this context by the significant influence of loading conditions; indeed, the frequent occurrence of vomiting and diarrhea in patients treated by chemotherapy determines load changes that could reduce the ability of GLS to assess subtle LV systolic dysfunction, resulting in the overestimation of myocardial damage and leading to unjustified therapy discontinuation.

**Figure 7:**
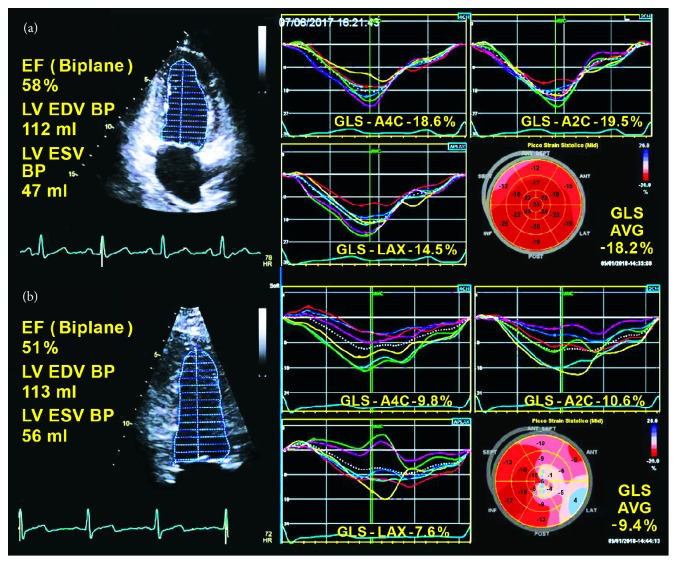
Assessment of left ventricular systolic function through two-dimensional Simpson's method and global longitudinal strain curves with bull's-eye plots in two patients undergoing chemotherapy with mild (a) and severe (b) left ventricular dysfunction. Please note that in the first case (a) ejection fraction is normal (58%) whereas global longitudinal strain is mildly impaired (-18.2%) with some regional areas of early damage and that, in the second case (b), although the ejection fraction is only at low limits (51%), global longitudinal strain is severely reduced (-9.4%) with an extensive regional impairment, as shown in the bull's-eye plots.

Bull's-eye maps show a frequent involvement of the septum and, often, an apical impairment (Figure 7; Table 3). However, these data need a more robust confirmation before they can be considered in the clinical practice.

Finally, we agree with the current scientific documents that GLS with bull's-eye map evaluation is indicated for an early detection of cardiac toxicity and to monitor cardiac effects of cancer therapy in long-term surveillance programs as well.

## 8. Conclusive Authors' Opinions

The growing evidence of the superiority of GLS as a marker of global function compared with conventional analysis for an early diagnosis of myocardial damage and for risk stratification is, in our opinion, the key role of this tool in each clinical setting. Additional information provided by bull's-eye maps suggests that regional LS has the potential to improve the accuracy of clinical diagnosis according to specific patterns. However, as with all echocardiographic parameters, GLS is only one piece of the puzzle; this should be kept uppermost in mind to avoid overstating its usefulness. Regional LS still suffers from some limitations, and thus, currently, it could be reasonable and more useful to consider regional LS distribution as a semiquantitative tool, focusing attention on the assessment of differences between segments in bull's-eye maps to find typical patterns that can help obtain the correct diagnosis rather than evaluating the numerical segment-specific strain values.

In the near future, improvements in technology could allow wider use of longitudinal strain analysis in the everyday routine, offering a better diagnostic accuracy and reliability and, consequently, an increased role in patient management (Supplementary Table 2).

## Figures and Tables

**Table 1:** Hot topics of two-dimensional longitudinal strain: cautions.

(i) Stroke volume and heart rate dependency
(ii) Age, race, and gender differences
(iii) Intervendor variability
(iv) Image quality dependency
(v) Interobserver and intraobserver variability
(vi) High technical skill requirement
(vii) Necessity of high frame rate
(vii) Pre-load and after-load dependency
(ix) Influence from medications, hemodynamics, and volume status
(x) Little diagnostic specificity

**Table 2:** Usefulness of global and regional longitudinal strain in clinical settings.

GLS	Regional LS
(i) Prediction of outcomes in patients with ICM, HCM, and HFpEF (ii) Prediction of adverse LV remodeling and poor outcomes after MI (iii) Stratification of risk profile and improvement of timing for surgery in asymptomatic patients with severe AS, AR, or MR and normal standard parameters of LV function (iv) Early detection of cancer therapeutics-related cardiac dysfunction	(i) Differential diagnosis of LVH (ii) Detection of myocardial fibrosis in patients with HCM and aortic stenosis (iii) Diagnosis of cardiac amyloidosis (iv) Differential diagnosis between anterior MI and takotsubo syndrome (v) Detection of MI site and size (vi) Detection of acute coronary occlusion in NSTEMI patients (vii) Detection of scar regions in ischemic patients (viii) Identification of areas of myocardial edema in myocarditis

AS: aortic stenosis; AR: aortic regurgitation; EF: ejection fraction; HCM: hypertrophic cardiomyopathy; HFpEF: heart failure with preserved ejection fraction; ICM: ischemic cardiomyopathy; LV: left ventricle; LVH: left ventricular hypertrophy; MI: myocardial infarction; NSTEMI: non-ST-elevation MI.

**Table 3:** Common patterns of regional distribution of longitudinal strain alterations.

Cardiac disease	Regional distribution of longitudinal strain alterations
Athlete's heart	Lower basal strain and more pronounced base-to-apex gradient
Hypertrophic cardiomyopathy	Specific patterns according to left ventricular hypertrophy distribution (septal, apical, diffuse)
Fabry disease	Lower strain of basal posterolateral wall
Amyloidosis	Impairment of basal and mid segments with normal function of apical segments (“apical sparing”)
Myocardial infarction	According to the vessel involved, reflects the coronary artery disease distribution
Takotsubo cardiomyopathy	Typical “circumferential pattern” involving all mid segments and clearly depicting the apical ballooning
Aortic stenosis	Impairment of basal segments of the anterior wall and septum
Duchenne muscular dystrophy	Impairment of inferolateral segments
Cardiotoxicity	Impairment of basal segments of the anterior wall and septum and/or apical involvement

## Supplementary Materials

Supplementary Materials Supplemental Figure 1: reduction of myocardial deformation in a patient with HFpEF. Supplemental Figure 2: longitudinal strain analysis in severe aortic stenosis. Supplemental Figure 3: global longitudinal strain bull's-eye maps depicting different patterns of left ventricular systolic dysfunction according to etiology. Supplemental Figure 4: global longitudinal strain bull's-eye maps in a patient with Duchenne muscular dystrophy with the involvement of the inferolateral wall. Supplemental Table 1: advantages and disadvantages of GLS with bull's-eye plot analysis. Supplemental Table 2: future direction of longitudinal strain analysis.
