# Supplementary material for: Ten Years of 2D Longitudinal Strain for Early Myocardial Dysfunction Detection: A Clinical Overview
Source: Biomed Res Int. 2018 Dec 5;2018:8979407. doi: 10.1155/2018/8979407 (PMC6304576; doi:10.1155/2018/8979407)
Supplement: Supplementary Materials — Supplemental Figure 1: reduction of myocardial deformation in a patient with HFpEF. Supplemental Figure 2: longitudinal strain analysis in severe aortic stenosis. Supplemental Figure 3: global longitudinal strain bull's-eye maps depicting different patterns of left ventricular systolic dysfunction according to etiology. Supplemental Figure 4: global longitudinal strain bull's-eye maps in a patient with Duchenne muscular dystrophy with the involvement of the inferolateral wall. Supplemental Table 1: advantages and disadvantages of GLS with bull's-eye plot analysis. Supplemental Table 2: future direction of longitudinal strain analysis. [file 8979407.f1.doc]

**Supplemental Material**

**Ten Years of 2D Longitudinal Strain for Early Myocardial Dysfunction Detection: A Clinical Overview**

**
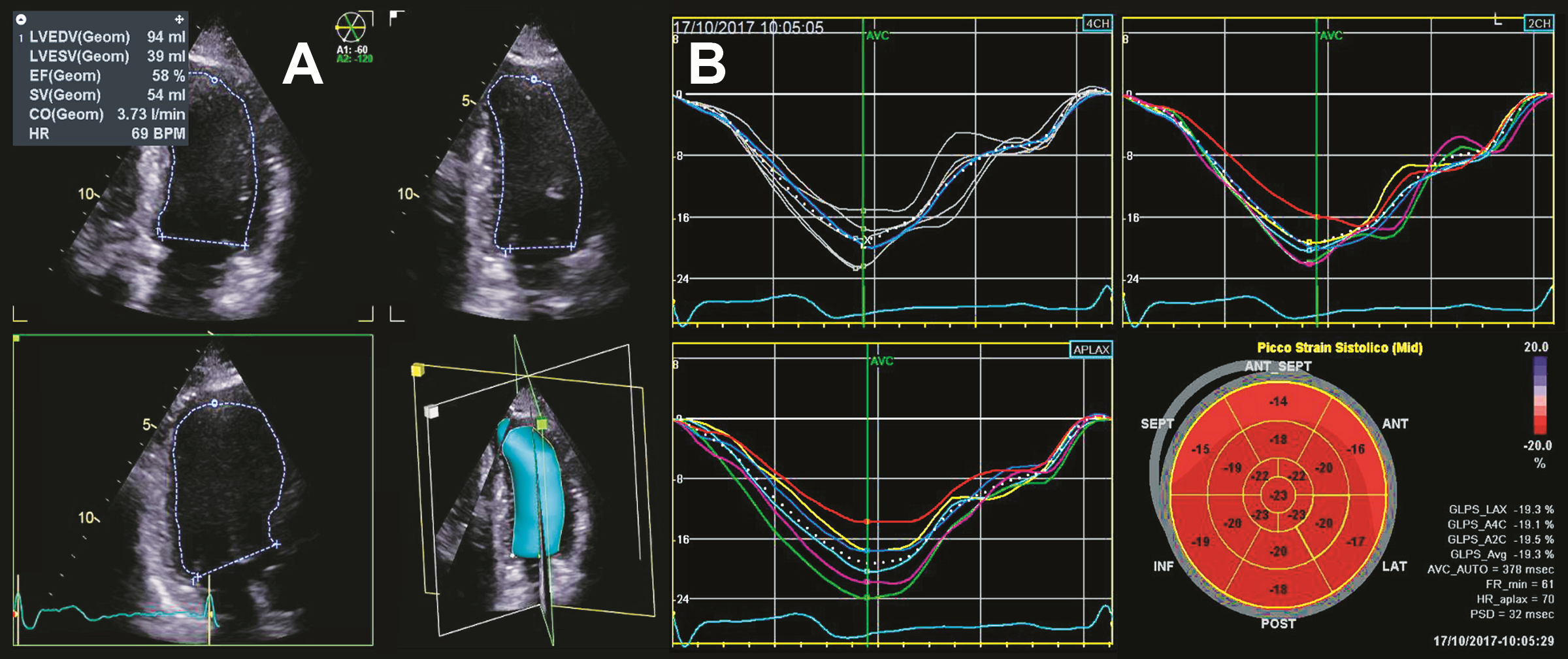
**

**Supplemental Figure 1.** Echocardiographic assessment of left ventricular systolic function in a patient with heart failure with preserved ejection fraction through three-dimensional triplane ejection fraction **(A)** and two-dimensional speckle-tracking echocardiography global longitudinal strain curves and bull’s-eye map **(B)** showing a reduction of myocardial deformation at the basal segments of the septum and anterior wall.

**
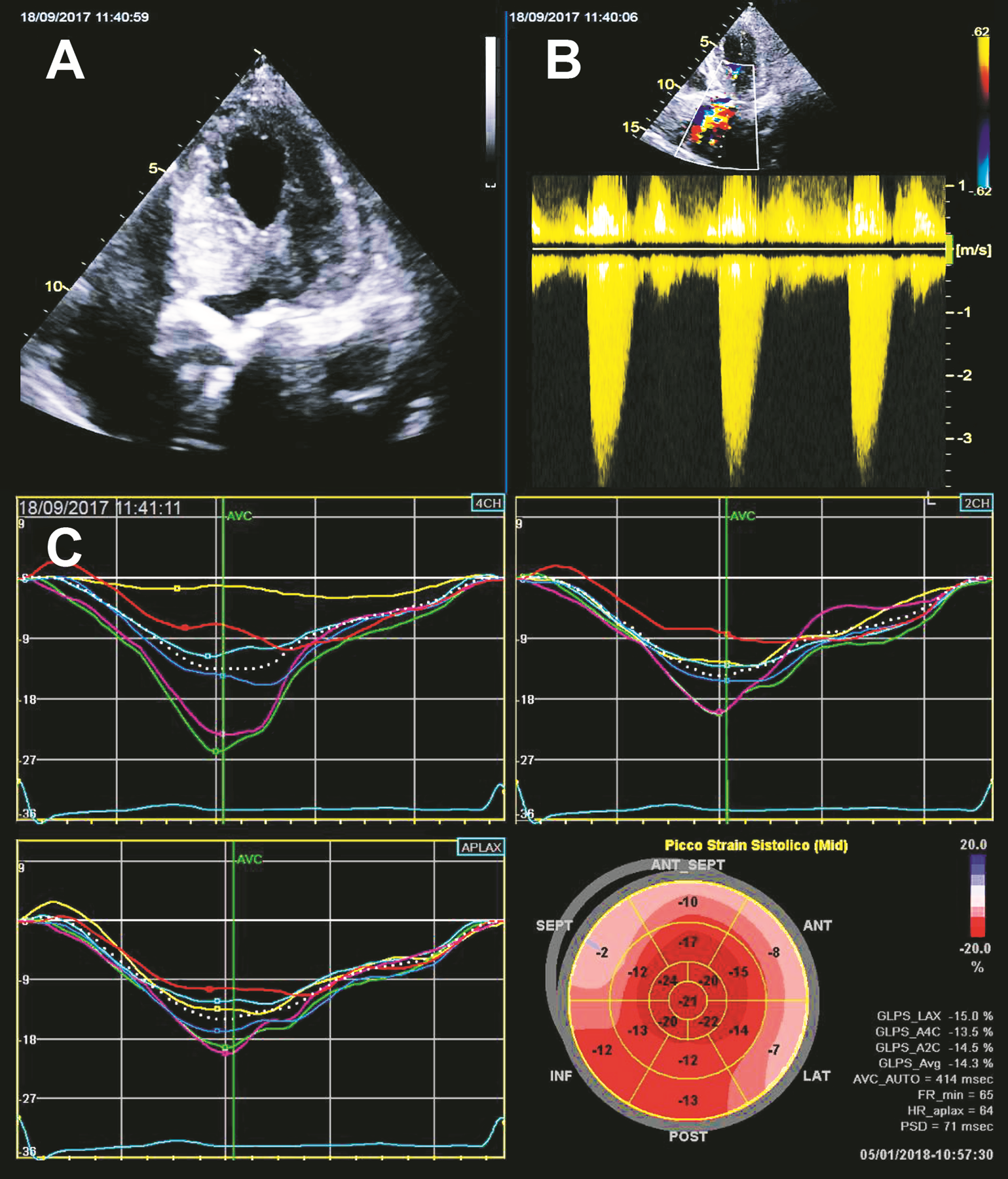
**

**Supplemental Figure 2.** A 59-year-old patient with severe aortic stenosis. **Panel A** shows a severe left ventricular hypertrophy more evident in the basal segments. **Panel B** shows increased aortic valve gradient and velocity assessed by continuous-wave Doppler. **Panel C** shows left ventricular two-dimensional speckle-tracking echocardiography longitudinal strain curves and bull’s-eye map: please note that basal segments have more impaired longitudinal function than apical ones.

**
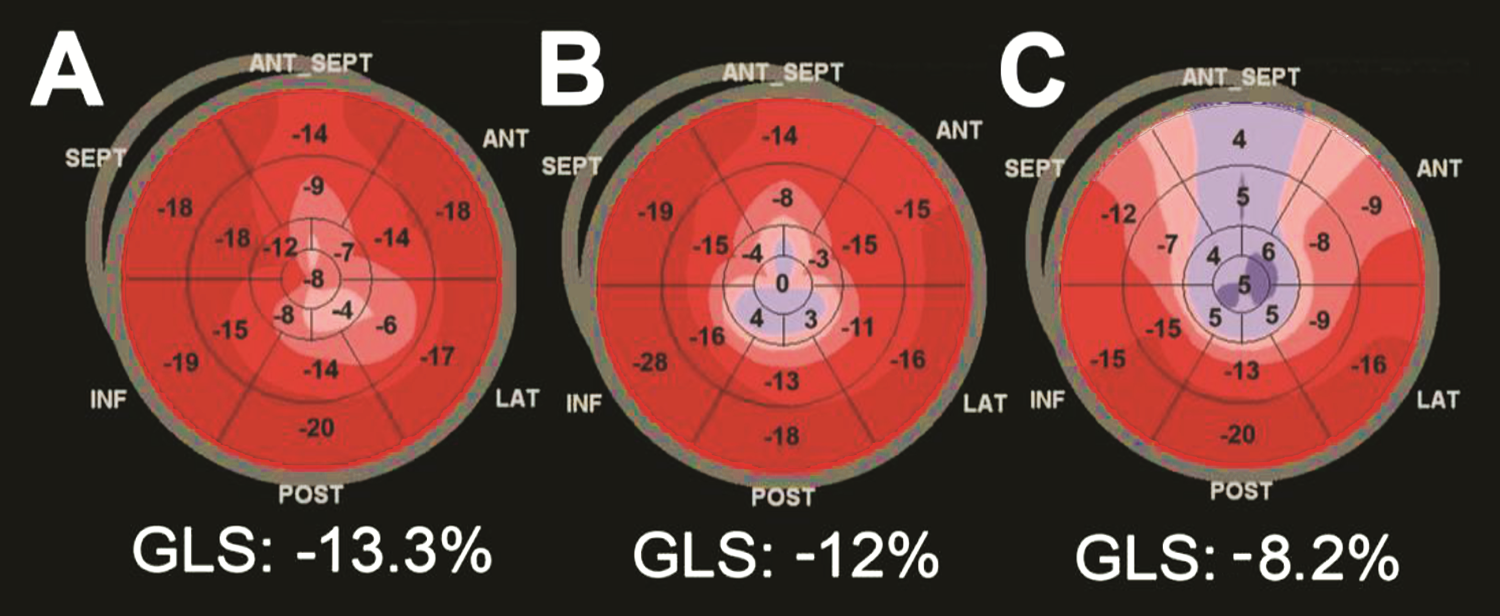
**

**Supplemental Figure 3.** Global longitudinal strain bull’s-eye maps depicting different patterns of left ventricular systolic dysfunction according to etiology. **(A)** Myocarditis with an extension of damage that does not reflect any coronary distribution. **(B)** Takotsubo syndrome with the typical “circumferential pattern” involving all mid-segments depicting (blue segments) the apex ballooning. **(C)** Anterior myocardial infarction with strain impairment of proximal left anterior descending artery corresponding left ventricle segments.

**
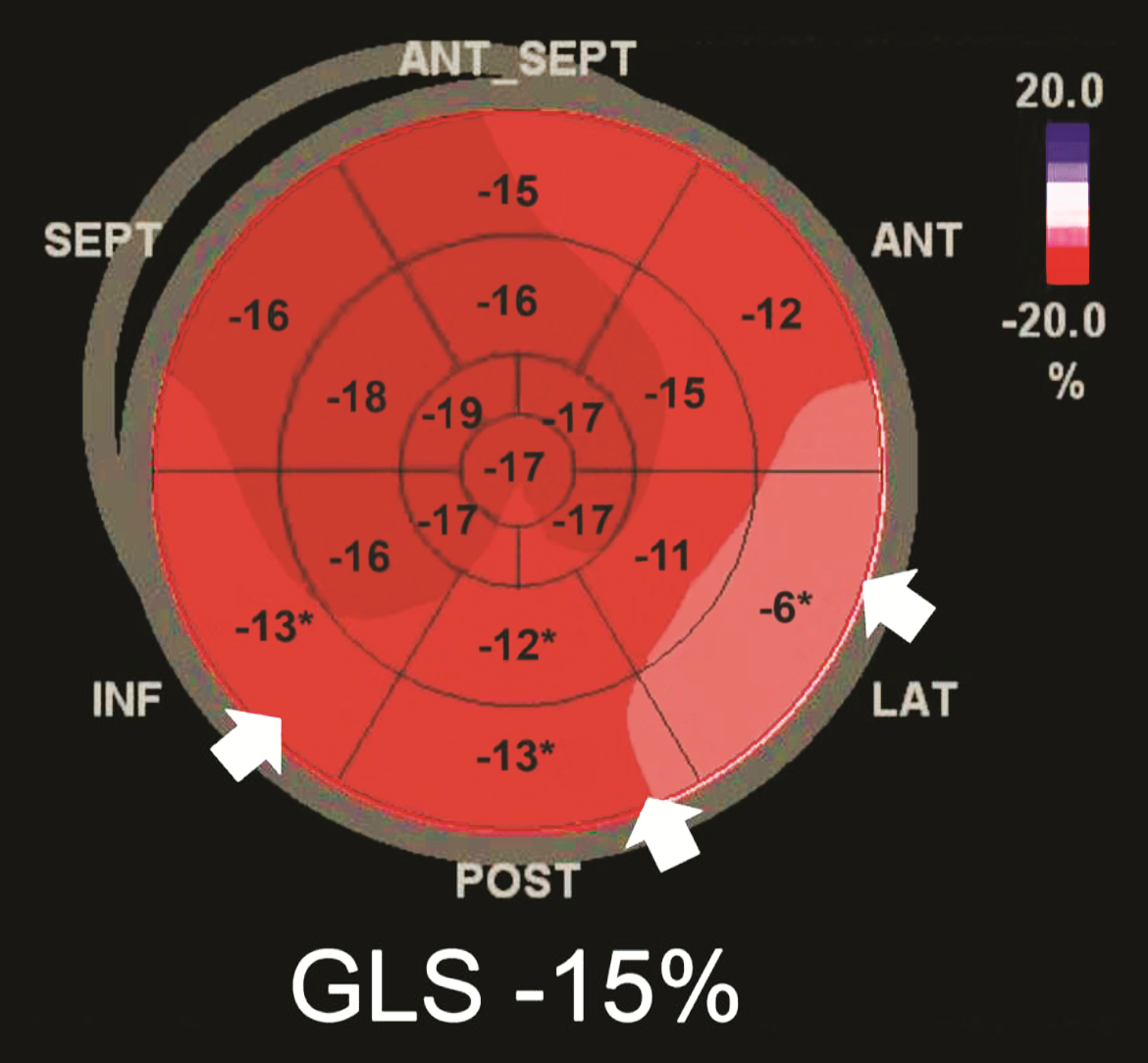
**

**Supplemental Figure 4.** Global longitudinal strain bull’s-eye maps in a patient with Duchenne muscular dystrophy with the involvement of the inferolateral wall **(white arrows)**.

**Supplementary Table 1.** GLS with bull’s-eye plot analysis: advantages, disadvantages, and authors’ recommendations for clinical use

|  | **Advantages** |  | **Disadvantages*** | **Recommendation** |
| --- | --- | --- | --- | --- |
| Left ventricle hypertrophy | | | | |
| - Differential diagnosis between physiological and pathological LVH and among all causes of pathological LVH - Early diagnosis of subclinical systolic dysfunction before LV geometric remodeling - Myocardial fibrosis “mapping” (excellent diagnostic power in comparison with MRI) - Identification of HCM patients at high risk of arrhythmias (role of mechanical dispersion) - Predictor of outcome in HFPEF | | | - Possible regional alterations with normal GLS - Not useful after septal ablation in HCM - Limitations from comorbidities inducing LVH | - Strongly indicated |
| Ischemic cardiomyopathy | | | | |
| Acute coronary syndrome | | | - Non-optimal accuracy in distinguishing transmural from subendocardial necrosis - Limitations of regional strain - Low weight in decision-making of patients with ACS | - Very useful |
| - Differential diagnosis with takotsubo and myocarditis - Accurate detection of infarct size and area of necrosis - Early diagnosis of myocardial ischemia and systolic dysfunction - Stress echocardiography: better accuracy for CAD detection than wall motion - Prognostic power for CV event occurrence | | |  |  |
| Chronic ischemic cardiomyopathy | | |  | - Very useful |
| - Prognostic relevance (GLS and mechanical dispersion) | | |  |  |
| Valvular heart disease | | | | |
| Aortic stenosis | | | - Possible regional alterations with normal GLS - Limitations from comorbidities inducing LVH or LV remodeling - Influenced by stroke volume - Load dependency | - Indicated with caution |
| - Early detection of subtle contractile dysfunction - Identification of typical patterns of basal fibrosis - High prognostic power in asymptomatic patients and in LFLG-AS - Prediction of long-term outcomes and mass regression after AVR - Prediction of prognosis after TAVR | | |  |  |
| Aortic regurgitation | | |  | - Useful |
| - Detection of early myocardial dysfunction in asymptomatic patients - Prediction of outcomes during conservative management and after surgery - Early detection of the progression of AR in the young | | |  |  |
| Mitral regurgitation | | | |  |
| - Detection of early myocardial dysfunction in asymptomatic patients - Optimization of surgical timing and prediction of LV dysfunction and HF after MV repair - Prediction of CIMR recurrence after repair | | | - Not useful in mild primary MR - Limited utility in CIMR | - Very useful |
| Systemic disease, metabolic and neuromuscular disorders | | | | |
| - Diagnosis of preclinical systolic dysfunction and adverse LV remodeling - Prediction of CV events during follow-up | | | - Influenced by other coexisting disease | - Very useful |
| Cardiotoxicity | | | | |
| - Early diagnosis of myocardial dysfunction in patients with normal EF - Typical pattern of alterations (septal and apical dysfunction) | | | - Need for the same equipment for longitudinal follow-up - Influence of loading conditions | - Indicated |

*Poor specificity and need for optimal image quality for all conditions.

ACS, acute coronary syndrome; AR, aortic regurgitation; AVR, aortic valve replacement; CAD, coronary artery disease; CIMR, chronic ischemic mitral regurgitation; CV, cardiovascular; EF, ejection fraction; GLS, global longitudinal strain; HCM, hypertrophic cardiomyopathy; HF, heart failure; HFPEF, heart failure with preserved ejection fraction; LFLG-AS, low-flow, low-gradient aortic stenosis; LV, left ventricle; LVH, left ventricle hypertrophy; MR, mitral regurgitation; MRI, magnetic resonance imaging; MV, mitral valve; TAVR, transcatheter aortic valve replacement.

**Supplementary Table 2.Future direction of longitudinal strain analysis**

| - Reduction of differences between vendors - Wide spread of strain imaging and improvement of echocardiographers’ skills for its correct use - Increasing role of regional distribution of longitudinal strain alterations for differential diagnosis of cardiovascular diseases, with the increase of the specificity of the measure - New fields of application of strain analysis (e.g., role of spatial heterogeneity and temporal dyssynchrony of strain distribution for the risk stratification of patients eligible for ICD implantation, etc.) - Improve 3-dimensional strain accuracy and reproducibility - Use of dedicated strain software for the assessment of right ventricle and atria and/or simultaneous strain of the heart - Developing software for cardiac principal strain analysis that is able to overcome multidirectional strain assessment |
| --- |

ICD, implantable cardioverter defibrillator.
